# Cancer-associated fibroblasts: multifaceted regulators of the tumor microenvironment and emerging therapeutic targets

**DOI:** 10.3389/fimmu.2026.1990436

**Published:** 2026-10-26

**Authors:** Jinsong Yu, Han Yang, Xiaofang Zhu

**Affiliations:** 1 Center for Plastic & Reconstructive Surgery, Department of Lymphatic Surgery and Reconstructive Microsurgery, Zhejiang Provincial People’s Hospital, Affiliated People’s Hospital, Hangzhou Medical College, Hangzhou, Zhejiang, China; 2 Department of Endocrinology, Nanyang Nanshi Hospital, Nanyang, Henan, China; 3 Center for General Practice Medicine, Department of Nursing, Zhejiang Provincial People’s Hospital, Affiliated People’s Hospital, Hangzhou Medical College, Hangzhou, Zhejiang, China

**Keywords:** cancer-associated fibroblasts, fibroblast heterogeneity, phenotypic plasticity, stromal reprogramming, stromal targeting, tumor microenvironment

## Abstract

Cancer-associated fibroblasts (CAFs) are a heterogeneous population of nonmalignant stromal cells that influence tumor progression and treatment response. They can arise from resident fibroblasts and other progenitor populations, with their relative contributions varying across tumor types and experimental settings. CAF states and functions are shaped by their cellular origins, local signals, spatial niches, and therapeutic pressure. CAFs participate in extracellular matrix remodeling, immune regulation, metabolic interactions, and angiogenesis, with effects that can promote or restrain tumor progression depending on tumor type and local conditions. This review summarizes CAF origins, molecular states, functional diversity, and signaling networks. We assess therapeutic strategies involving CAF depletion, extracellular matrix modification, signaling disruption, and phenotypic normalization, distinguishing preclinical findings from clinical outcomes. The clinical benefit of these approaches requires further validation in defined patient populations. Finally, we discuss how single-cell and spatial profiling and patient-derived models may improve CAF characterization and inform the development of selective stromal interventions.

## Graphical Abstract

**Figure:**
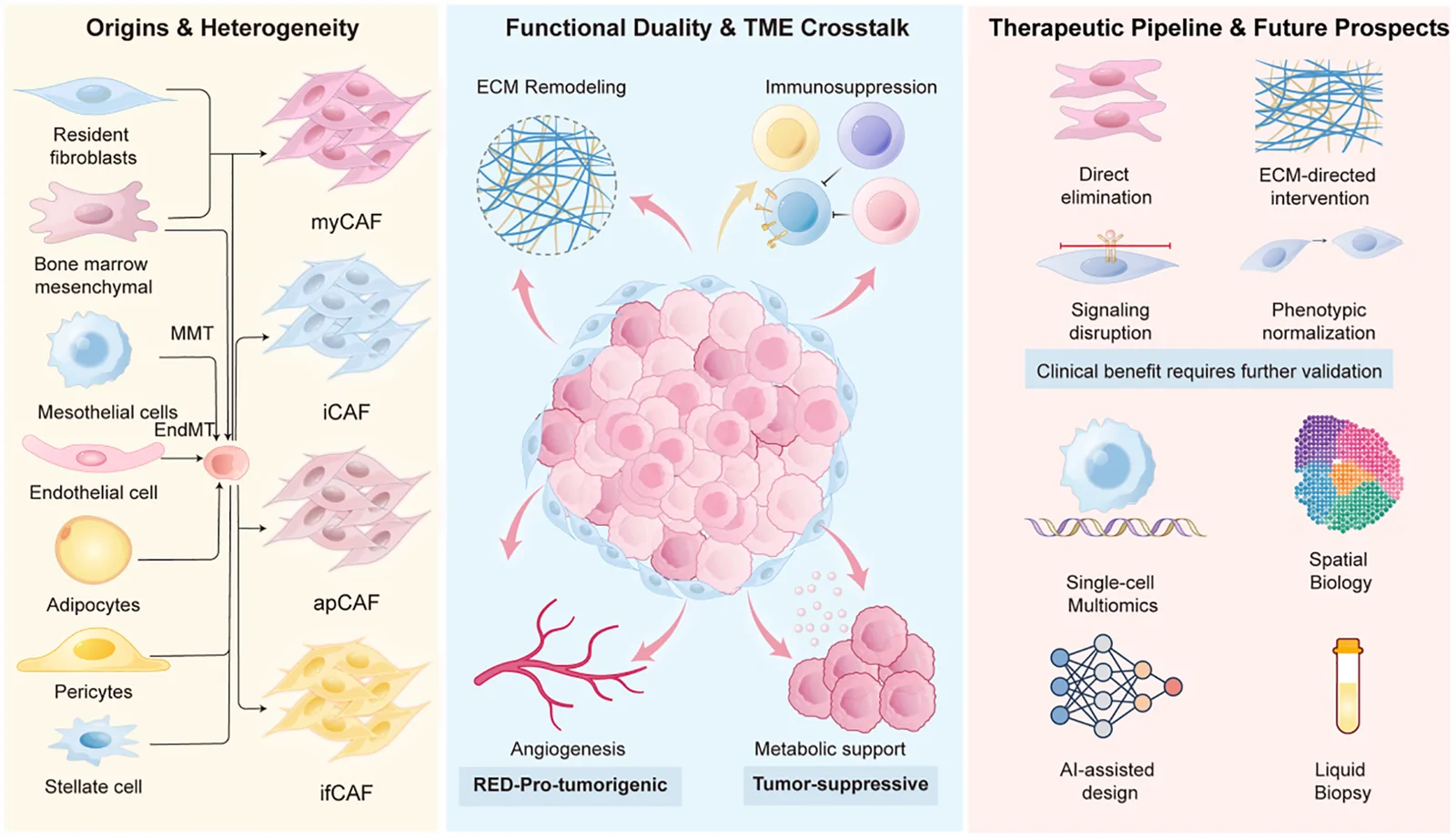

## 1 Introduction

### 1.1 Historical perspective: from passive scaffold to active orchestrator

The tumor microenvironment (TME) is a complex ecosystem where malignant cells interact dynamically with various non-tumor components (1, 2). Among these stromal components, cancer-associated fibroblasts (CAFs) have gradually become the focus of cancer research over the past two decades. Researchers believe that they are important contributors to tumor progression, immune evasion, and treatment resistance. The biological concept of CAFs has evolved from viewing fibroblasts as passive components that merely provide a collagen structural scaffold to active and multifunctional participants involved in various aspects of malignant tumors (3, 4).

This change in perspective first emerged in the late 1990s to the early 21st century. Previous studies suggested that CAFs could directly promote the progression of already initiated prostate epithelial tumors *in vivo* (5). A subsequent study showed that CAFs in the breast can promote angiogenesis and tumor growth by increasing the secretion of CXCL12 (6). These groundbreaking discoveries established that fibroblasts in these experimental settings could actively support the development of malignant tumors. CAF populations can express markers such as fibroblast activation protein (FAP), α-smooth muscle actin (α-SMA), platelet-derived growth factor receptor alpha (PDGFRα), podoplanin (PDPN), and fibroblast-specific protein 1 (FSP1/S100A4) (7–11). However, none of these markers is universally expressed by CAFs or exclusive to them. CAF identification therefore requires a combination of markers, tissue localization, and exclusion of malignant and other non-fibroblast cell populations.

### 1.2 The paradigm shift: heterogeneity and functional duality

Over the past decade, the most significant breakthrough in CAF research has been the recognition of the heterogeneity of CAFs. The application of single-cell RNA sequencing (scRNA-seq) technology has played a crucial role in revealing this complexity. In studies targeting pancreatic ductal adenocarcinoma (PDAC) (12), non-small cell lung cancer (13), breast cancer (14), colorectal cancer (15) and other malignant tumors, researchers have all discovered multiple coexisting CAF subtypes within the tumors. Each subtype, while sharing consistent characteristics, also has unique transcriptional programs and functional specialization features. People have gradually realized that different CAF subtypes may have opposite effects (16, 17). These effects are not necessarily intrinsic to a defined CAF population but may vary with tumor type, disease stage, spatial location, signaling, and treatment exposure. This understanding has informed the design of CAF-directed therapeutic strategies. In mouse PDAC models, elimination of α-SMA^+^ myofibroblasts was found to accelerate disease progression (18). This functional duality suggests that we need to adopt more detailed and precise methods for targeting the stroma.

### 1.3 What this review covers

This review focuses on three core themes: (1) the cellular origin and molecular heterogeneity of CAFs; (2) the various tumor-promoting and tumor-suppressing functions of CAFs; (3) emerging therapeutic strategies and their clinical translation. We emphasize how technological advancements have reshaped our understanding of CAFs and provided ideas for therapeutic interventions. Throughout the article, we distinguish cellular origin from molecular state and functional effects under different biological and treatment conditions, and assess the potential and limitations of treatment approaches targeting CAFs.

## 2 Origins and heterogeneity of CAFs

### 2.1 Cellular lineage diversification

CAFs exhibit significant functional heterogeneity, which is partly attributed to their diverse cell origins. CAFs can be generated through the differentiation of multiple precursor cell populations in specific environments. Understanding this lineage diversity is crucial for interpreting the functional heterogeneity of CAFs and for designing therapeutic strategies (**Figure 1**).

**Figure 1:**
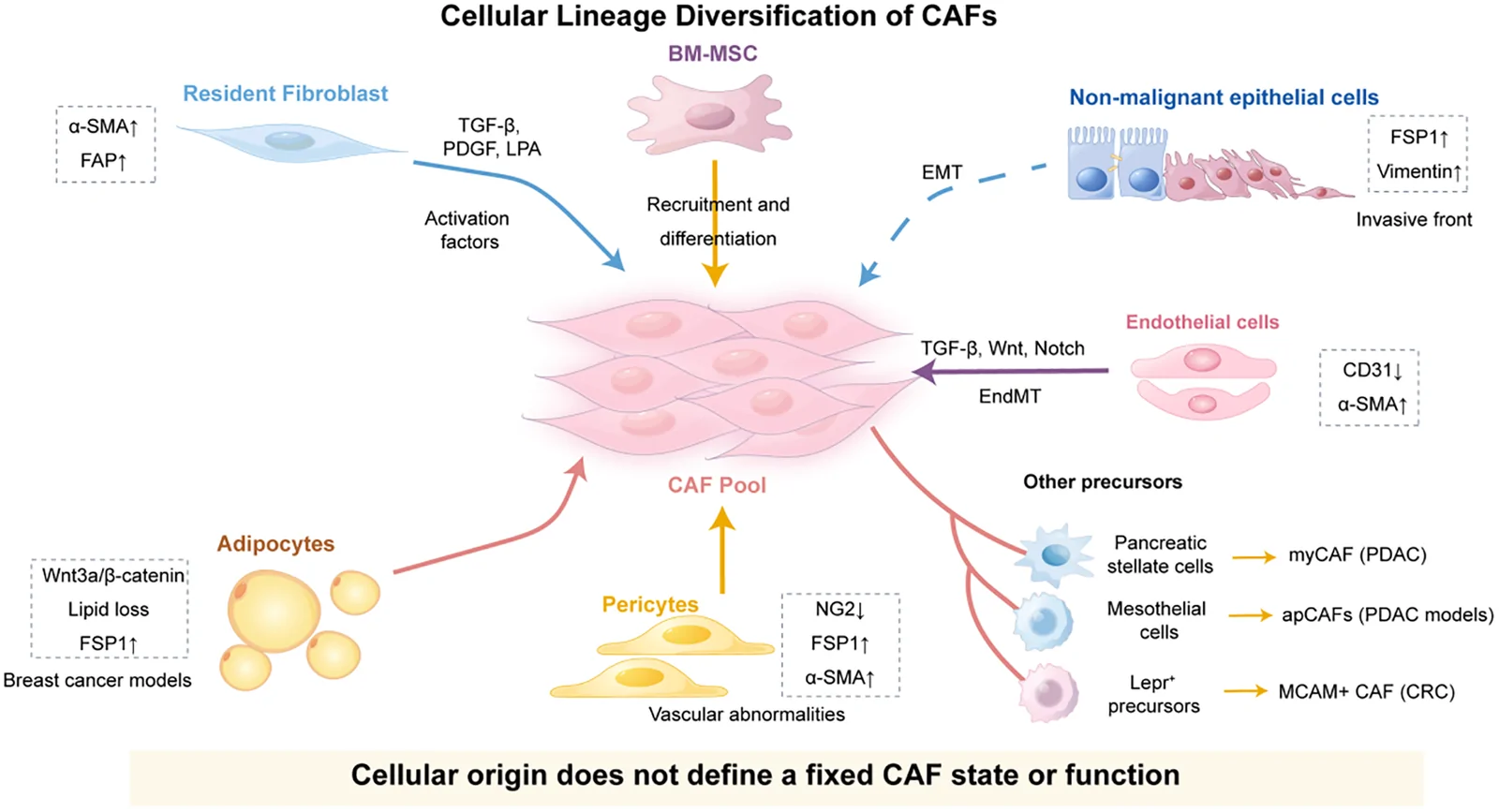
The origin of CAFs. Representative CAF progenitor populations and associated signals are shown across tumor models. The dashed arrow denotes the proposed contribution of nonmalignant epithelial cells through EMT. Upward and downward arrows indicate increased and decreased marker expression, respectively. apCAF, antigen-presenting CAF; BM-MSC, bone marrow-derived mesenchymal stem cell; CAF, cancer-associated fibroblast; CD31, cluster of differentiation 31; CRC, colorectal cancer; EMT, epithelial-to-mesenchymal transition; EndMT, endothelial-to-mesenchymal transition; FAP, fibroblast activation protein; FSP1, fibroblast-specific protein 1; Lepr, leptin receptor; LPA, lysophosphatidic acid; MCAM, melanoma cell adhesion molecule; myCAF, myofibroblastic CAF; NG2, neural/glial antigen 2; PDAC, pancreatic ductal adenocarcinoma; PDGF, platelet-derived growth factor; TGF-β, transforming growth factor β; α-SMA, α-smooth muscle actin.

#### 2.1.1 Resident fibroblast activation

CAFs can originate from the resident fibroblasts present in tumor-bearing tissues, although their relative contribution varies across tumor types and experimental models. Under normal physiological conditions, these cells maintain tissue homeostasis by regulating the production of the extracellular matrix (ECM). However, in the TME, continuous exposure to factors derived from cancer cells (such as transforming growth factor-β (TGF-β), platelet-derived growth factor (PDGF), and lysophosphatidic acid (LPA)) activates downstream signaling pathways, leading to the formation of CAF phenotypes (19–21). This activation process involves transcriptional and epigenetic reprogramming, often accompanied by the upregulation of α-SMA, FAP, or various collagen genes depending on the CAF state (22–24). In experimental mouse models, CAFs have been shown to mainly originate from local precursors rather than from precursor cells recruited from the circulatory system (25). The activation process of CAFs is driven by multiple interacting signaling pathways. The TGF-β family ligands can activate the transcription mediated by SMAD2/3, while LPA can promote the expression of α-SMA through serum response factor-associated pathways (26–28). The increasingly stiff tumor stroma further enhances this activation process through YAP-dependent mechanotransduction, thereby forming a positive feedback loop to maintain the activated phenotype (29).

#### 2.1.2 Recruitment and differentiation of mesenchymal stem cells

Bone marrow-derived mesenchymal stem cells (BMSCs) constitute a potential progenitor source for CAFs in specific tumor models. In mouse models of inflammation-induced gastric cancer, tumor-derived signals recruit MSCs to the tumor site, where the local microenvironment drives their differentiation into CAF-like cells (30). This CAF-like differentiation has been demonstrated in experimental models of prostate and breast cancer (31–33).

In human neuroblastoma, researchers identified a population of CAFs that simultaneously expresses FAP and FSP-1. This population shares phenotypic and functional characteristics with BMSCs, but these similarities do not establish a bone marrow origin (34). The relative contribution of MSC-derived versus resident fibroblast-derived CAFs may vary by tumor type, anatomical location, and disease stage, contributing to the observed inter- and intra-tumoral CAF heterogeneity.

#### 2.1.3 Potential contribution of nonmalignant epithelial cells

Epithelial cells can acquire mesenchymal features through EMT, a developmental program frequently co-opted during cancer progression. During this process, the epithelial cells lose their polarity, cell-cell adhesion, and epithelial markers, while acquiring mesenchymal characteristics, including the expression of FSP1 and fibronectin (35, 36). However, malignant epithelial cells undergoing EMT remain cancer cells and should not be classified as CAFs solely on the basis of mesenchymal marker expression (28). The contribution of non-malignant epithelial cells to the CAF pool remains an area of active investigation. Establishing this contribution requires lineage-resolved evidence that excludes malignant cells; marker expression or transcriptional similarity alone is insufficient. This proposed route should also be distinguished from mesothelial-to-mesenchymal transition.

#### 2.1.4 Endothelial-to-mesenchymal transition

Endothelial cells represent another potential CAF progenitor population in some tumor models. Through EndMT, endothelial cells downregulate characteristic markers such as CD31 and VE-cadherin while acquiring fibroblast-associated markers including FSP1 and α-SMA (37–39). This transition can be driven by TGF-β1, Wnt, and Notch signaling, depending on the experimental conditions (40–42). In glioblastoma, EndMT-associated CAF-like cells have been implicated in ECM deposition and temozolomide resistance through TNC- and FLNC-associated mechanisms (43).

#### 2.1.5 Adipocyte conversion

Adipocytes constitute an alternative progenitor reservoir for CAFs, a source particularly relevant in tumors arising within or adjacent to adipose-rich environments such as breast cancer and peritoneal metastases of gastric cancer (44). Tumor-derived Wnt3a activates β-catenin signaling in adipocytes in breast cancer models, driving their conversion into adipocyte-derived fibroblasts (ADFs) characterized by FSP1 upregulation, loss of lipid droplets, and acquisition of a fibroblastic morphology (45). These ADFs actively participate in ECM remodeling within the TME through increased secretion of fibronectin and collagen I (45). In breast cancer, ADFs are present at the invasive front. Their association with enhanced local invasion highlights the functional importance of adipose-derived stromal cells in tumor progression within the adipose microenvironment (45).

#### 2.1.6 Pericyte-to-CAF transition

Pericytes are vascular cells embedded in the basement membrane of capillaries. Under tumor-associated PDGF-BB stimulation, they can detach from the vascular structure and undergo a transition toward a fibroblast-like phenotype in experimental models (46). The resulting loss of pericyte coverage can contribute to vascular abnormalities and increased permeability, fostering a microenvironment conducive to tumor cell intravasation and hematogenous dissemination (46). The effects of anti-PDGF treatment on pericyte coverage and vascular remodeling depend on tumor PDGF-BB expression (47).

#### 2.1.7 Additional progenitor populations

Emerging evidence has identified further CAF progenitor sources of considerable importance. Pancreatic stellate cells (PSCs) represent a source of myofibroblastic CAFs in PDAC, where they contribute to the characteristic desmoplastic reaction (48–50). However, lineage tracing identified PSC-derived CAFs as a numerically minor subset with distinct matrix-related functions in a PDAC mouse model, indicating that their quantitative contribution should not be generalized across experimental settings (51). In response to TGF-β signaling, PSCs can undergo significant phenotypic transformation, converting into α-SMA^+^ myofibroblast-like CAFs; IL-1 instead favors an inflammatory program through LIF–JAK/STAT signaling in experimental PDAC systems (19, 22).

In mouse models of colorectal cancer, the pericryptal stromal precursor cells of the leptin receptor (Lepr) lineage proliferate and differentiate into MCAM^+^ CAFs. These cells promote macrophage recruitment and tumor growth (52). Mesothelial cells can undergo mesothelial-to-mesenchymal transition to generate antigen-presenting CAFs (apCAFs) under the regulation of IL-1/TGF-β signaling in PDAC models (53). Mesothelial-to-mesenchymal transition has also been implicated in peritoneal metastases of ovarian cancer (54, 55).

The recognition of this diverse cellular ancestry suggests that CAF heterogeneity reflects, at least in part, distinct cellular origins. However, cellular origin does not establish a fixed molecular state or functional role; these are also shaped by microenvironmental signals and tumor biology (56).

### 2.2 Molecular heterogeneity and CAF states

The application of high-resolution profiling techniques, particularly single-cell RNA sequencing, spatial transcriptomics and proteomics analysis, has enabled the systematic mapping of the heterogeneity of CAFs in malignant tumors. Several recurrent CAF states have been identified across multiple cancer types, although nomenclature, defining markers, and functional annotations vary between studies (**Table 1**; **Figure 2**). These molecular states are not necessarily mutually exclusive, and their functional effects cannot be inferred from marker expression alone.

**Table 1:** Representative CAF states and their functional properties.

Reported functional effects	Subtype	Representative markers or features	Primary function	Open question
Context-dependent	myCAFs	α-SMA, POSTN, TAGLN	ECM deposition and remodeling, and generation of contractile force. Matrix effects can support invasion or restrain tumor growth, depending on matrix composition and experimental context (16, 18, 22).	Does α-SMA^+^ cell depletion help or harm in non-PDAC cancers?
Tumor-supportive in PDAC models	iCAFs	IL-6, LIF, CXCL1; lower α-SMA than myCAFs in PDAC	Inflammatory cytokine secretion and paracrine support of tumor-cell survival; the inflammatory program is promoted by IL-1–LIF–JAK/STAT signaling (19, 22)	Can inflammatory signaling be inhibited while preserving beneficial stromal functions?
Context-dependent	apCAFs	MHC-II, CD74	MHC-II-mediated antigen presentation to CD4^+^ T cells; Treg expansion in PDAC models or support of CD4^+^ effector T-cell activity in lung cancer models (12, 53, 57).	Which patients benefit from apCAF expansion versus depletion?
Tumor-restraining in PDAC models	ifCAFs	Upregulated interferon-stimulated genes (ISGs)	Interferon-response program; reduced tumor-cell invasion and promotion of an antitumor phenotype in tumor-associated neutrophils in PDAC models (58).	Can STING agonists be combined safely with ICIs?

Representative molecular features and functional effects are summarized for the indicated tumor settings. CAF states can overlap and change with local signals.

apCAF, antigen-presenting CAF; CAF, cancer-associated fibroblast; CD, cluster of differentiation; CXCL1, C-X-C motif chemokine ligand 1; ECM, extracellular matrix; iCAF, inflammatory CAF; ICI, immune checkpoint inhibitor; ifCAF, interferon-responsive CAF; IL, interleukin; ISG, interferon-stimulated gene; JAK, Janus kinase; LIF, leukemia inhibitory factor; MHC-II, major histocompatibility complex class II; myCAF, myofibroblastic CAF; PDAC, pancreatic ductal adenocarcinoma; POSTN, periostin; STAT, signal transducer and activator of transcription; STING, stimulator of interferon genes; TAGLN, transgelin; Treg, regulatory T cell; α-SMA, α-smooth muscle actin.

**Figure 2:**
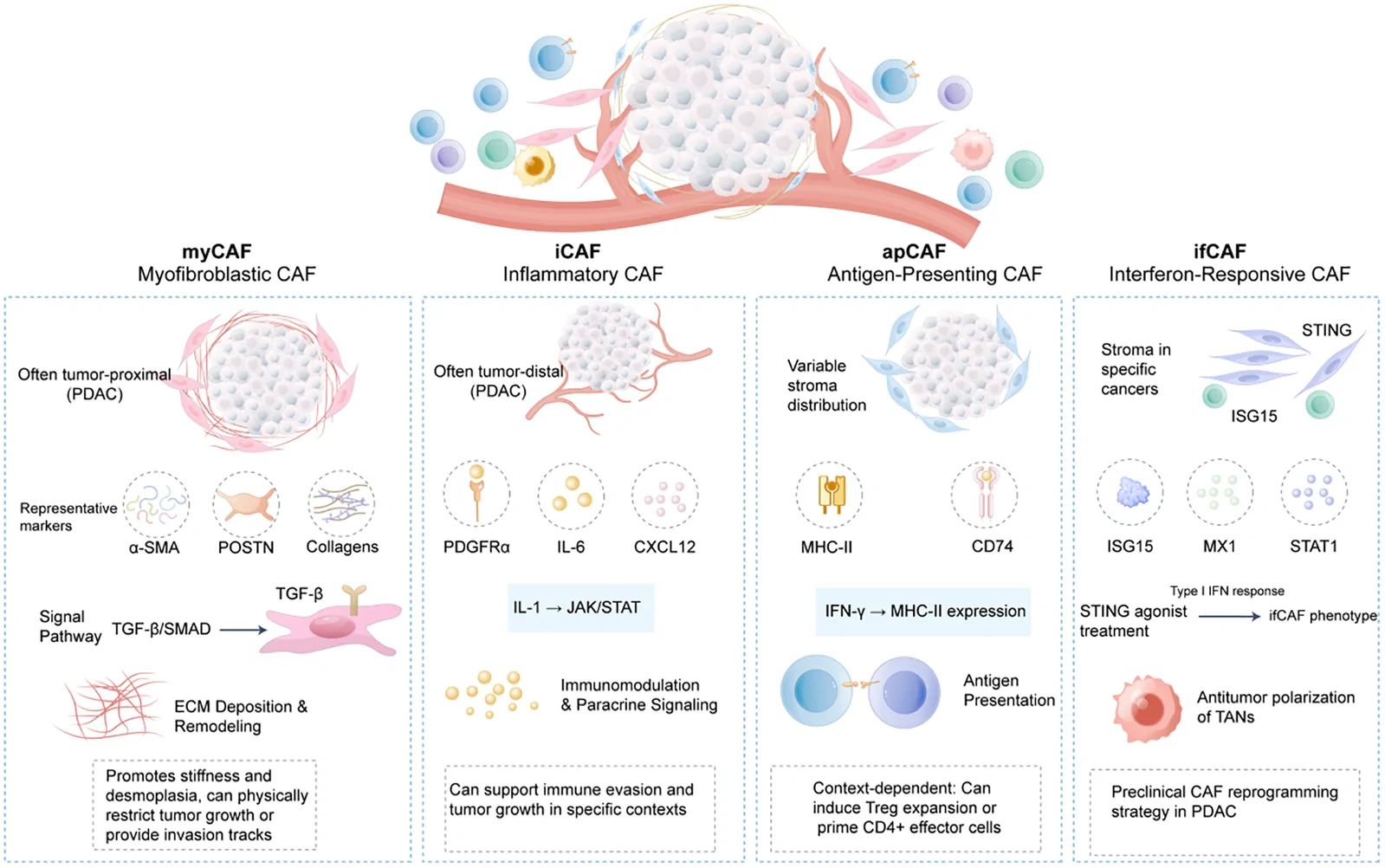
CAF subtypes and their functional specialization. CAF states are compared by spatial distribution, molecular features, signaling programs, and functional effects. MyCAF and iCAF localization is illustrated in PDAC, while apCAF functions include Treg expansion in PDAC and CD4^+^ effector responses in lung cancer. The ifCAF panel depicts STING-induced reprogramming and antitumor neutrophil polarization in PDAC models. apCAF, antigen-presenting CAF; CAF, cancer-associated fibroblast; CD, cluster of differentiation; CXCL12, C-X-C motif chemokine ligand 12; ECM, extracellular matrix; iCAF, inflammatory CAF; IFN, interferon; ifCAF, interferon-responsive CAF; IL, interleukin; ISG15, interferon-stimulated gene 15; JAK, Janus kinase; MHC-II, major histocompatibility complex class II; MX1, MX dynamin-like GTPase 1; myCAF, myofibroblastic CAF; PDAC, pancreatic ductal adenocarcinoma; PDGFRα, platelet-derived growth factor receptor α; POSTN, periostin; STAT, signal transducer and activator of transcription; STING, stimulator of interferon genes; TAN, tumor-associated neutrophil; TGF-β, transforming growth factor β; Treg, regulatory T cell; α-SMA, α-smooth muscle actin.

#### 2.2.1 Myofibroblastic CAFs

Among the known CAF subtypes, myCAFs have been extensively studied. These cells often express high levels of α-SMA, periostin (POSTN), transgelin (TAGLN), and various collagens in the models studied (22, 59–61). They are located in close proximity to the areas where cancer cells gather and exhibit highly contractile characteristics. This myofibroblastic program is promoted by the TGF-β/SMAD2/3 signaling pathway in experimental PDAC systems (19). A prominent function of myCAFs is to deposit collagen and other structural proteins, assemble them into a dense network, and generate tensile force, thereby reorganizing the structure of the matrix. The resulting desmoplastic stroma is a typical feature of many invasive cancers. It can affect the migration of tumor cells, the penetration of drugs, and the infiltration of immune cells. The relationship between myCAFs and tumor progression is not simple. The remodeled matrix can provide a path for tumor invasion and may also restrain tumor growth, depending on its composition and the tumor model. This functional variation is particularly evident when α-SMA^+^ myofibroblasts are experimentally depleted in PDAC mouse models. Depleting α-SMA^+^ myofibroblasts led to a more aggressive disease, characterized by invasive, poorly differentiated tumors and increased immunosuppression, with reduced survival (18).

#### 2.2.2 Inflammatory CAFs

iCAFs differ from myCAFs in their predominant transcriptional and secretory programs. iCAFs express PDGFRα and produce elevated levels of inflammatory cytokines and chemokines (IL-6, CXCL12, CCL2), although this profile varies with tumor type and local signals and is not specific to iCAFs (19, 62). In PDAC, their α-SMA levels are lower than those of myCAFs, and they tend to be distributed in regions distant from the cancer cell nests. iCAFs mainly exert their effects through paracrine signals. IL-6, CXCL12, and CCL2 have distinct effects on the migration of myeloid cells, the localization of T cells, and vascular biology (63, 64). The balance between myCAFs and iCAFs is dynamically regulated by competing environmental signals in experimental PDAC systems. TGF-β promotes a myofibroblastic state and inhibits the inflammatory program partly by downregulating IL-1R1, while IL-1 promotes an iCAF program through LIF-mediated activation of the JAK/STAT pathway (19). Reversible transitions between inflammatory and myofibroblastic states have been observed after changing PSC culture conditions (22). However, prolonged IL-1α exposure limited subsequent TGF-β1-induced myofibroblastic differentiation in prostate-derived MSCs, indicating that this plasticity depends on cellular state and experimental conditions (65).

#### 2.2.3 Antigen-presenting CAFs

Single-cell sequencing revealed the existence of CAF populations that express MHC II molecules (HLA-DR/DP/DQ) and CD74 (12). These cells can present antigens to CD4^+^ T cells through their MHC-II molecules in experimentally validated settings, thereby influencing the adaptive immune response (12, 57). In PDAC, mesothelial cells are one experimentally supported source of apCAFs. These cells undergo transformation through the mesothelial-to-mesenchymal transition process driven by IL-1 and TGF-β in the studied models (53). The specific role of apCAFs within the tumor remains an active research topic, and their functional activity is highly dependent on the specific microenvironment. In PDAC models, apCAFs facilitate the expansion of regulatory T cells and promote immunosuppression. In lung cancer models, apCAFs with interferon-γ-dependent MHC-II expression support the activation and effector functions of CD4^+^ T cells, thereby supporting anti-tumor immunity (57). Further studies have shown that apCAFs also exhibit heterogeneity. A cross-tissue single-cell and spatial analysis identified two major apCAF populations with mesothelial-like and fibrocyte-like features (66). These populations illustrate diversity within the apCAF category rather than a universal classification across all cancers. This molecular and spatial heterogeneity adds new challenges to clearly defining the functional roles of the CAF subgroups.

#### 2.2.4 Interferon-responsive CAFs

Interferon-responsive CAFs (ifCAFs) are characterized by the upregulation of interferon-stimulated genes (ISGs) (67, 68). In PDAC models, induction of an ifCAF phenotype suppressed tumor-cell invasion and promoted an antitumor phenotype in tumor-associated neutrophils (58). Studies have shown that STING agonists can induce this phenotype both *in vitro* and *in vivo*. This suggests that CAF state reprogramming may offer a preclinical therapeutic strategy without the need to eliminate CAFs (58). However, interferon responsiveness alone does not establish an antitumor function. In bladder cancer, an interferon-dependent SLC14A1^+^ CAF population promoted cancer stemness through WNT5A signaling (69). These populations should not be assumed to represent the same subtype across cancers.

#### 2.2.5 Additional CAF states and marker-defined populations

Beyond these commonly described states, numerous additional CAF populations have been described across cancer types. Senescent CAFs can exhibit a senescence-associated secretory phenotype (SASP). In gastric cancer models, inflammatory signals sustained this phenotype, and senescent CAFs enhanced peritoneal tumor formation through JAK/STAT3 signaling (70).

FAP^+^ CAFs represent a broadly defined population that encompasses multiple subtypes. FAP has emerged as a widely studied therapeutic target due to its expression on activated CAFs and its accessibility for therapeutic targeting, although it is not CAF-specific (71, 72). Meflin (ISLR)-positive CAFs are associated with improved clinical outcomes in PDAC and exhibit tumor-inhibitory properties in experimental models (73, 74). Lipid-loaded ABCA8a^+^ CAFs in pancreatic cancer models with tumor-cell Setd2 deficiency provide metabolic support to tumor cells through lipid transfer, sustaining oxidative phosphorylation (75). CD146 (MCAM)-positive fibroblast populations exhibit functions that vary across tumor types: in estrogen receptor-positive breast cancer models, they maintain hormone dependence and tamoxifen sensitivity, whereas in gastric cancer they promote M2 macrophage polarization and immunosuppression through COL4A1 (76, 77). These marker-defined populations and functional states can overlap, and their therapeutic relevance requires assessment for each tumor type and treatment setting.

### 2.3 Regulation of CAF heterogeneity

The heterogeneity of CAFs is dynamically regulated by various spatiotemporal factors. Among them, microenvironmental signals are important regulators. For instance, TGF-β promotes myofibroblastic programs, IL-1 promotes inflammatory programs, and interferon signals induce IFN-responsive phenotypes in experimental PDAC systems (19, 58). TGF-β can antagonize IL-1-driven inflammatory signaling, thereby influencing the balance between these states (19). Matrix mechanics and therapeutic interventions can also modify CAF programs, as illustrated by YAP-dependent mechanotransduction and the emergence of interferon-responsive fibroblasts after TGF-β blockade (29, 67). Additionally, epigenetic regulation plays a role in maintaining the characteristics and plasticity of CAF states. For example, reduced H3K27 methylation at the ADAMTS1 promoter was associated with increased ADAMTS1 expression in stromal fibroblasts exposed to breast cancer cells (24). Epigenetic alterations in neighboring tumor cells can also influence CAF states: tumor-cell SETD2 loss in pancreatic cancer models enhances BMP2 signaling and favors lipid-rich CAFs (75).

Moreover, the spatial organization within the tumor adds to its complexity, with different CAF states preferentially enriched in particular cellular neighborhoods (14, 78). This spatial organization can influence exposure to local signals and opportunities for cell interactions, thereby influencing CAF functions. In addition, the heterogeneity among tumors needs to be taken into account. Although myofibroblastic and inflammatory programs recur across various tumor types, these programs do not provide an exhaustive classification, and the relative abundance of CAF subtypes, their specific markers, and their functional roles vary across tumor types (14, 78).

## 3 Signaling mechanisms of CAF activation and CAF-tumor crosstalk

There is a complex two-way communication between cancer cells and CAFs, which can promote or restrain tumor progression depending on the cell populations involved and local signaling. We next examine the five widely studied signaling pathways (TGF-β/SMAD, Wnt/β-catenin, Hedgehog (Hh), Notch, and PDGF/PDGFR signaling pathways) (summarized in **Table 2**; **Figure 3**).

**Table 2:** Core signaling pathways in CAF-tumor communication.

Pathway	Effects on tumor cells and the TME	Pathway regulation in CAFs	Therapeutic caution
TGF-β → SMAD	EMT activation, invasion, metastasis in breast cancer models (79)	TGF-β1 → TβRII/TβRI → SMAD2/3 → SMAD4-containing complexes → target-gene transcription and CAF activation (80, 81)	Effects depend on the responding cell population and tumor context.
Wnt → β-catenin	Proliferation, invasion and stemness through CAF-derived exosomal miR-20a-5p/LIMA1 and miR-92a-3p/AXIN1 axes in hepatocellular carcinoma models; DKK1 from CAFs suppresses NK cells in breast cancer models (82–84)	Oxidized ATM-associated signaling involving Wnt/β-catenin contributes to breast CAF proliferation (85)	DKK1-mediated NK-cell suppression may occur independently of canonical Wnt signaling; Wnt inhibition should not be assumed to reverse this effect (84)
Hedgehog → GLI	Paracrine support: CAF-derived FGF5 and fibrillar collagen support stemness and chemoresistance in triple-negative breast cancer mouse models (86)	SHH → PTCH1 → relief of SMO inhibition → GLI activation → target-gene expression (87)	Short-term SMO inhibition reduced tumor growth in PDAC mouse models, while decreasing myCAFs, enriching iCAFs and increasing immunosuppression, with fewer CD8^+^ T cells and more Tregs (87)
Notch → NICD	Stemness through CAF-derived STC1–Notch1 signaling in hepatocellular carcinoma models; EMT and metastasis through CAF-derived COMP–Notch3 signaling in ovarian cancer models (88, 89)	Exposure to apoptotic lung cancer cells activates Notch1–WISP-1 signaling in existing CAFs (90)	Notch can also suppress metastasis (Notch1-WISP-1) in mouse lung cancer models (90)
PDGF/PDGFR	PDGF signaling stimulates CAF-derived FGF2 production, supporting angiogenesis in a cervical carcinoma mouse model (91)	PDGF-BB → PDGFRβ → IKK → NF-κB → α-SMA, FAP upregulation in cultured human oral mucosal fibroblasts (92)	PDGFR targeting also affects pericytes and vascular remodeling; treatment effects depend on tumor PDGF-BB expression and experimental context (47)

Pathways are summarized by their regulation within CAFs, effects on tumor and immune cells, and therapeutic implications. Arrows indicate signaling sequences.

ATM, ataxia telangiectasia mutated; CAF, cancer-associated fibroblast; COMP, cartilage oligomeric matrix protein; DKK1, Dickkopf-1; EMT, epithelial-to-mesenchymal transition; FAP, fibroblast activation protein; FGF, fibroblast growth factor; iCAF, inflammatory CAF; IKK, IκB kinase; LIMA1, LIM domain and actin-binding protein 1; miR, microRNA; myCAF, myofibroblastic CAF; NF-κB, nuclear factor κB; NICD, Notch intracellular domain; NK, natural killer; PDAC, pancreatic ductal adenocarcinoma; PDGF, platelet-derived growth factor; PDGFR, platelet-derived growth factor receptor; PTCH1, Patched1; SHH, Sonic Hedgehog; SMO, Smoothened; STC1, stanniocalcin-1; TβRI/TβRII, type I/type II TGF-β receptor; TGF-β, transforming growth factor β; TME, tumor microenvironment; Treg, regulatory T cell; WISP-1, Wnt1-inducible signaling pathway protein 1; α-SMA, α-smooth muscle actin.

**Figure 3:**
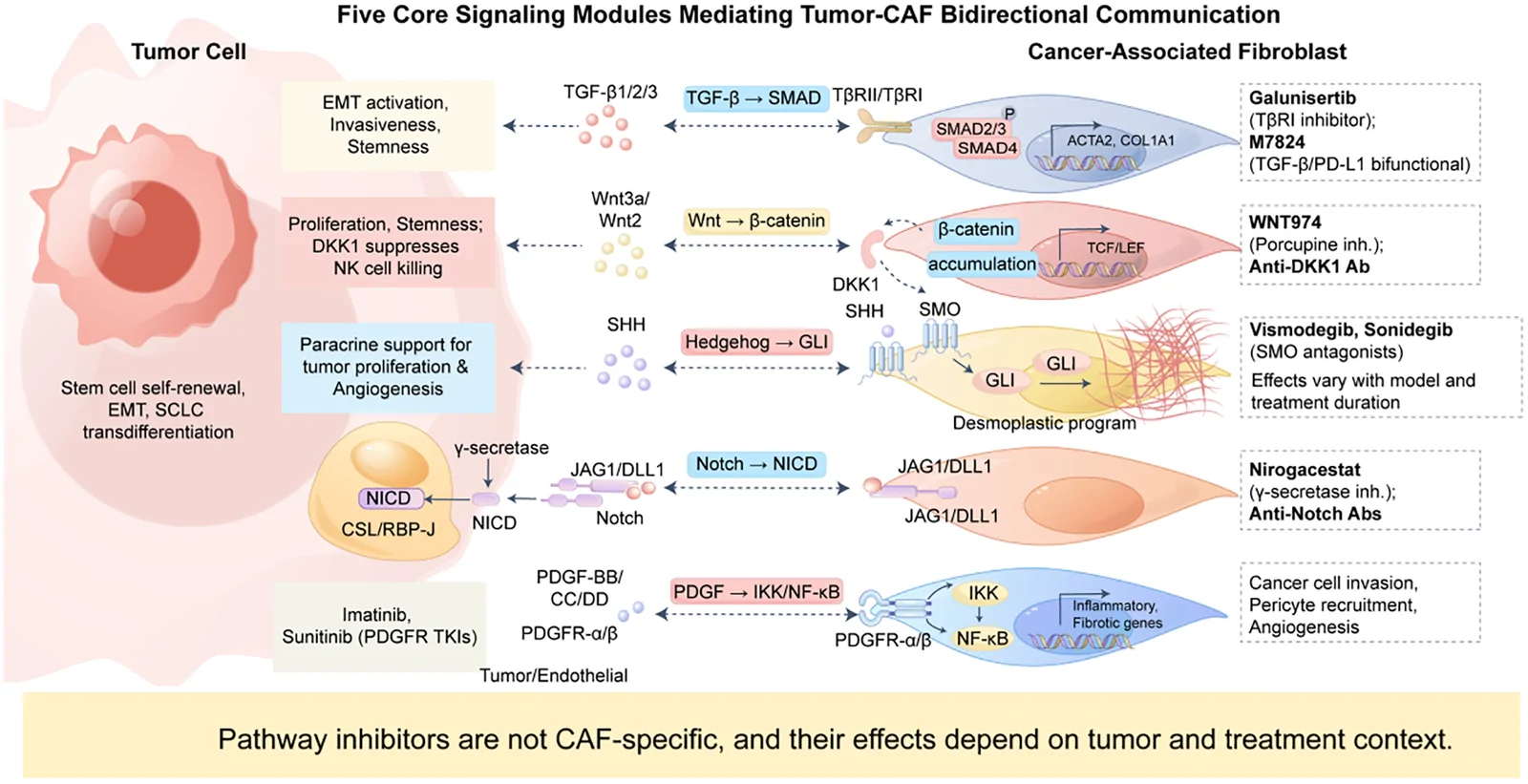
Five core signaling pathways involved in CAF activation and CAF-tumor crosstalk. TGF-β, Wnt, Hedgehog, Notch, and PDGF signaling connect CAF activation with tumor-cell behavior and stromal remodeling. Representative ligands, intracellular mediators, cellular effects, and pathway inhibitors are shown. P denotes phosphorylation. Ab, antibody; ACTA2, actin α2, smooth muscle; CAF, cancer-associated fibroblast; COL1A1, collagen type I α1 chain; CSL, CBF1/Suppressor of Hairless/LAG-1; DKK1, Dickkopf-1; DLL1, Delta-like ligand 1; EMT, epithelial-to-mesenchymal transition; IKK, IκB kinase; JAG1, Jagged1; NF-κB, nuclear factor κB; NICD, Notch intracellular domain; NK, natural killer; PDAC, pancreatic ductal adenocarcinoma; PDGF, platelet-derived growth factor; PDGFR, platelet-derived growth factor receptor; PD-L1, programed death-ligand 1; RBP-J, recombination signal binding protein for immunoglobulin κ J region; SCLC, small cell lung cancer; SHH, Sonic Hedgehog; SMO, Smoothened; TCF/LEF, T-cell factor/lymphoid enhancer-binding factor; TβRI/II, type I/II TGF-β receptor; TGF-β, transforming growth factor β; TKI, tyrosine kinase inhibitor.

### 3.1 TGF-β/SMAD signaling

The TGF-β signaling pathway is one of the most extensively studied axes during the activation of CAFs. The TGF-β1 secreted by tumors can bind to the type II TGF-β receptor (TβRII) on fibroblasts. After ligand binding, TβRII recruits and phosphorylates TβRI, which further phosphorylates SMAD2 and SMAD3. The phosphorylated SMAD2/3 proteins form a complex with SMAD4 and translocate to the nucleus, thereby activating the transcription of target genes (80, 93). Additionally, activated CAFs can secrete TGF-β and further enhance this signaling pathway, forming a positive feedback loop for CAF activation and maintaining the myofibroblast-like phenotype in mammary tumor models (81). The stiffness of the extracellular matrix can facilitate contraction-dependent activation of TGF-β1 from the latent complex, as shown in myofibroblast studies, supporting a positive feedback loop between biochemical and biomechanical signals (94).

In parallel, the TGF-β produced by CAFs can promote epithelial-mesenchymal transition (EMT), invasion and metastasis after acting on cancer cells through specific receptors. In breast cancer cells, the SMAD2/3/4 complex can transcriptionally activate the long non-coding RNA HOTAIR, contributing to EMT and metastasis (79). Moreover, the TGF-β signaling pathway also intersects with epigenetic regulation within CAFs. In breast CAFs, TET1-mediated demethylation of the SMAD4 promoter enhances SMAD4 expression and establishes a positive feedback loop through GATA6, which promotes the transcription of TGF-β in CAFs (95). This pathway can exert anti-tumor effects by inhibiting epithelial cell proliferation in some settings, while promoting invasion and immune evasion in others; these effects depend on the responding cell population, tumor type, and disease stage (93, 96). These opposing effects complicate the clinical translation of TGF-β inhibitors.

### 3.2 Wnt/β-catenin signaling

The Wnt/β-catenin signaling pathway can regulate the behavior of CAFs and their interactions with cancer cells. In breast CAFs, oxidized ATM promotes cell proliferation through signaling networks involving PI3K-AKT, MEK-ERK, and Wnt/β-catenin (85). Additionally, CAFs can also secrete Wnt antagonists, including Dickkopf-1 (DKK1). The secreted DKK1 inhibits the cytotoxicity of NK cells and promotes immune escape in breast cancer models by negatively regulating the phosphorylation of AKT/ERK/S6, apparently independently of canonical Wnt signaling (84).

The regulation of the Wnt signaling pathway through extracellular vesicles (EVs) is an emerging research direction. Exosomes derived from CAFs contain miR-20a-5p, which can target LIMA1 and activate the Wnt/β-catenin signaling pathway in cancer cells, thereby promoting growth and invasion in experimental hepatocellular carcinoma models (82). Similarly, CAF-derived exosomal miR-92a-3p suppresses AXIN1 and activates β-catenin signaling in hepatocellular carcinoma cells, promoting tumor growth and stemness (83).

### 3.3 Hedgehog signaling

The Hedgehog (Hh) signaling pathway mediates interactions between CAFs and tumors. The Sonic Hedgehog (SHH) ligand secreted by tumors can bind to the Patched (PTCH1) receptor on CAFs. By relieving the inhibition on Smoothened (SMO), it further activates GLI transcription factors and promotes the expression of target genes (87, 97). The inhibition of the Hh pathway produces complex effects. In PDAC mouse models, pharmacological Hh inhibition using the SMO inhibitor sonidegib (LDE225) reduces the number of myCAFs. At the same time, it increases the proportion of iCAFs, accompanied by fewer CD8^+^ T cells and increased Treg cell infiltration, consistent with increased immunosuppression (87). Short-term treatment also reduced tumor growth in the studied KPC model. This observation indicates that the intervention of Hh signaling may change the composition of CAF states and immune populations, with divergent effects on tumor growth and antitumor immunity.

### 3.4 Notch signaling

CAFs can influence multiple aspects of cancer progression through interactions involving the Notch signaling pathway. In hepatocellular carcinoma models, the stanniocalcin-1 (STC1) derived from CAFs can activate the Notch1 signaling pathway in cancer cells. It achieves this by promoting the translocation of the Notch intracellular domain (NICD) to the nucleus and maintaining the characteristics of cancer stem cells through an amplifying stromal-tumor signaling loop (88). In ovarian cancer models, the cartilage oligomeric matrix protein (COMP) secreted by CAFs can activate the Notch3 pathway by enhancing Notch3-Jagged1 interactions, thereby promoting epithelial-mesenchymal transition (EMT) and metastasis (89). In small cell lung cancer co-culture models, the IL-6 derived from lung fibroblasts can activate the Notch pathway through the JAK2/STAT3/c-MYC pathway, promoting the transition from neuroendocrine to non-neuroendocrine states and contributing to drug resistance (98).

However, under certain conditions, the Notch pathway may also have an anti-tumor effect. Studies have shown that exposure to apoptotic cancer cells can activate the Notch1-WISP-1 signaling pathway in existing CAFs, thereby inhibiting lung cancer metastasis in mice (90). This context-dependent regulation highlights the highly environment-dependent nature of the Notch pathway in CAF biology, with outcomes depending on the responding cell population and local signals.

### 3.5 PDGF/PDGFR and chemokine signaling

The activation of CAFs and the remodeling of the interstitium can involve the PDGF signaling pathway mediated by PDGFR-α/β. PDGF-BB binds to PDGFR-β on fibroblasts, which can activate the IKK/NF-κB signaling pathway and upregulate the expression of α-SMA and FAP, as shown in cultured human oral mucosal fibroblasts through a mechanism involving LURAP1L-AS1/LURAP1L (92). Additionally, the PDGF signal also supports angiogenesis by recruiting pericytes and stimulating FGF-2 production by CAFs in a mouse model of cervical carcinoma (91). The CXCL12-CXCR4 chemokine axis plays an important role in CAF-mediated immune regulation. Specifically, in a genetically engineered mouse model of PDAC, FAP^+^ CAFs are an important source of CXCL12. This factor can limit the accumulation of T cells among cancer cells and help promote immune checkpoint inhibitor resistance (7). Using AMD3100 (plerixafor) to block CXCR4 enhanced T cell infiltration and acted synergistically with anti-PD-L1 treatment in this model.

## 4 The multifaceted functions of CAFs in cancer progression

CAFs exert their influence on the development of cancer through a variety of interrelated mechanisms. These functions can be discussed in terms of tumor-promoting and tumor-suppressing activities. However, these activities do not define fixed CAF populations and may vary with tumor type, disease stage, spatial localization, local signaling, and treatment exposure.

### 4.1 Protumorigenic functions

#### 4.1.1 ECM remodeling and biomechanical signaling

CAFs are major producers of the ECM. They can secrete a large amount of structural components such as collagen (types I, III and V), fibronectin and tenascin C, and also produce ECM-modifying enzymes (99, 100). This combination of synthesis, crosslinking and degradation can produce a remodeled matrix microenvironment, characterized by increased stiffness, altered arrangement of collagen fibers, changes in ECM components, and increased interstitial fluid pressure (99).

The biomechanical outcomes of extracellular matrix remodeling affect multiple aspects of cell behavior. Tumor tissue stiffness can be greater than that of corresponding normal tissue (101). This increased stiffness can activate mechanotransduction pathways — including integrin-mediated FAK/SRC signaling, YAP/TAZ nuclear translocation, and Rho GTPase activation — that can promote cancer cell proliferation, invasion, EMT, and stemness with effects that vary by cell type and state (102). Importantly, matrix stiffness itself regulates CAFs activation, creating a self-amplifying loop wherein CAF stiffen the matrix, which in turn further activates CAFs.

Clinical studies have correlated elevated matrix stiffness with adverse outcomes. In papillary thyroid carcinoma, the increased peri-tumoral stiffness is associated with CAF activation, collagen deposition and lymph node metastasis (103). In colorectal cancer, the mean overall survival time for patients with high-stiffness tumors was significantly shorter than that of patients with low-stiffness tumors in a cohort of 107 patients (101).

CAF-mediated ECM remodeling also can facilitate cancer cell invasion through at least three interrelated mechanisms: (1) protease-dependent ECM degradation creating physical channels for invasion; (2) force-mediated physical reorganization of the matrix through actomyosin contractility; and (3) alignment of collagen fibers providing directional guidance for cell invasion (102, 104, 105). Additionally, CAFs may contribute to the establishment of pre-metastatic niches in distant organs through secreted factors and stromal remodeling (106, 107).

#### 4.1.2 Immunosuppressive niche formation

CAFs can be central orchestrators of the immunosuppressive TME. They can establish both physical and chemical barriers that impede CD8^+^ T cell infiltration and function, thereby limiting antitumor immunity and conferring resistance to immune checkpoint inhibitors (108–110). The inverse correlation between CAFs abundance and CD8^+^ tumor-infiltrating lymphocyte density has been documented across multiple cancer types and is associated with immunotherapy resistance and poor prognosis.

T cell exclusion: In a genetically engineered PDAC mouse model, FAP^+^ CAFs are the main source of CXCL12, which contributes to the exclusion of T cells from regions containing cancer cells (7). NOX4 activity in CAFs supports myofibroblastic activation and ECM production by generating reactive oxygen species, and NOX4 inhibition reduces CD8^+^ T-cell exclusion in CAF-rich mouse tumors (111, 112). Additionally, TGF-β signaling promotes T cell exclusion and limits effector differentiation in colorectal cancer models (96). The combined effect of these physical and chemical barriers makes CAF-mediated T cell exclusion an obstacle to effective immunotherapy. Induction and expansion of Treg cells: Fibroblasts that present antigens in PDAC models can induce the differentiation of naive CD4^+^ T cells into FoxP3^+^ regulatory T cells through antigen-specific MHC-II-mediated antigen presentation (53). Separately, α-SMA^+^ CAFs can form immunological synapses with existing Tregs and promote their activation and proliferation in an antigen- and autophagy-dependent manner (113).

Myeloid cell modulation: Beyond T cell modulation, CAFs can influence myeloid cell biology. In hepatocellular carcinoma and non-small cell lung cancer models, CAFs promote M2-like polarization of tumor-associated macrophages (114, 115).

In triple-negative breast cancer, CAFs promote monocyte recruitment through the CXCL12-CXCR4 signaling pathway and can induce monocyte differentiation into immunosuppressive STAB1^+^TREM2^high^ lipid-associated macrophages (LAMs) in co-culture; this LAM population was enriched in patients who were resistant to immune checkpoint inhibitors (116). CAFs also promote CXCL16-mediated monocyte recruitment in triple-negative breast cancer (117). In hepatocellular carcinoma, POSTN^+^ CAFs recruit macrophages and increase their SPP1 expression through IL-6/STAT3 signaling (118). Other mechanisms include the secretion of amyloid beta peptide, which induces the formation of neutrophil extracellular traps (NETs) through a ROS-dependent pathway in mouse tumor models (119), and CLCF1-mediated stimulation of CXCL6 and TGF-β secretion by hepatocellular carcinoma cells, which promotes the recruitment and N2-like polarization of tumor-associated neutrophils in hepatocellular carcinoma models (120).

#### 4.1.3 Metabolic reprogramming and nutrient crosstalk

CAFs engage in extensive metabolic crosstalk with cancer cells, a rapidly expanding area of investigation. CAFs can supply metabolic substrates that sustain tumor cell proliferation under nutrient-limited conditions, functioning as metabolic “helpers” within the TME (121, 122). In pancreatic cancer models, lipid-rich ABCA8a^+^ CAFs transfer lipids to tumor cells, supporting oxidative phosphorylation (OXPHOS) in SETD2-deficient tumors (75). This lipid transfer creates metabolic dependence of tumor cells on CAFs, representing a potential therapeutic vulnerability.

Through the ACSS2-SP1-SAT1 axis, acetate derived from CAFs can promote the survival of pancreatic cancer cells. Acetate is converted into acetyl coenzyme A by ACSS2, which then drives histone acetylation and SP1 acetylation, increasing SP1 stability and SAT1 expression to regulate polyamine metabolism (122). This pathway is particularly important under conditions of acidosis. CAFs lacking glutamine in pancreatic cancer models take up extracellular proteins by activating macropinocytosis. These proteins are then degraded and the amino acids are released, thereby supporting the survival of CAFs and cancer cells (123). CAF-derived exosomes containing METTL3 stabilize SLC7A5 mRNA through m^6^A modification, enhancing glutamine metabolism in non-small cell lung cancer cells (124).

CAFs regulate tumor glycolysis through FAK-dependent signaling, with FAK-depleted CAFs exhibiting altered chemokine profiles that enhance glycolysis via CCR1/CCR2-PKA signaling in cancer cells (121). CAF-derived exosomes containing LINC01711 promote glycolysis in breast cancer cells through miR-4510/NELFE axis activation (125), while CAF-secreted WNT5A upregulates hexokinase 2 (HK2) to enhance glycolysis in gastric cancer cells (126).

CAF-derived EVs serve as mediators of metabolic crosstalk, delivering functional cargo including proteins, circular RNAs, and long non-coding RNAs to tumor cells (106, 127, 128). In colorectal cancer, CAF-EVs can establish a self-reinforcing positive feedback loop: EVs deliver circTAX1BP1, which can upregulate the secretion of TGF-β in tumor cells by promoting VIRMA lactylation and m^6^A-dependent stabilization of SP1 mRNA, thereby activating ITGA11^+^ myCAFs, causing them to secrete more circTAX1BP1-containing EVs (129). Additionally, the proteins on the surface of CAFs-EVs further amplify the TGF-β signaling pathway to activate CAFs and inhibit the function of CD8^+^ T cells (130).

#### 4.1.4 Angiogenesis and vascular remodeling

CAFs can contribute to tumor angiogenesis through secretion of multiple pro-angiogenic factors. CXCL12, PDGF-C, WNT2 and WNT5A are examples of CAF-derived angiogenic mediators (6, 131–133). These factors can influence endothelial behavior and promote new blood vessel formation through distinct mechanisms.

FHL2-expressing CAFs promote endothelial tube formation *in vitro* and metastasis in lung adenocarcinoma models (134). FHL2 also supports CAF secretion of osteopontin, which contributes to cancer-cell migration. In hepatocellular carcinoma, single-cell and spatial transcriptomic analysis identified VEGFA^+^ fibroblasts within tumor regions and inferred VEGF-mediated interactions with capillary endothelial cells (135).

The CXCR4/CXCL12 axis promotes vascular permeability and tumor-cell intravasation in breast cancer models (136). Pharmacological inhibition of PDGFR signaling with imatinib suppressed invasive carcinoma progression and angiogenesis in a mouse model of cervical cancer, with reduced CAF-derived FGF-2 and decreased pericyte coverage (91).

#### 4.1.5 Therapy resistance

CAFs can contribute to resistance across multiple treatment modalities through diverse mechanisms that represent both a clinical challenge and a therapeutic opportunity. Chemotherapy resistance is promoted by CD10^+^GPR77^+^ CAFs, which sustain cancer stem cell properties through IL-8 and IL-6 secretion in breast and lung cancer models (137). The SPP1 secreted by CAFs can activate alternative signaling pathways such as the RAF/MAPK and PI3K/AKT pathways, thereby promoting resistance to sorafenib and lenvatinib in hepatocellular carcinoma models (138). In colorectal cancer models, COL8A1 secreted by THBS2^+^ CAFs promotes oxaliplatin resistance through PI3K/AKT-dependent EMT (139). Patients with recurrent EGFR-mutant lung adenocarcinoma containing PDPN^+^ CAFs exhibited a significantly reduced response rate to EGFR-TKIs, compared with those whose tumors contained PDPN-negative CAFs (140).

Radiotherapy resistance involves CAF-mediated enhancement of cancer cell survival through IL-6 signaling, which activates STAT3 in breast cancer models (141). CAF-derived HGF activates c-Met signaling in cancer cells, driving EMT and radioresistance in breast cancer models (142). CAF-secreted CXCL1 promotes ROS accumulation after irradiation and DNA damage response activation, fostering radioresistance in esophageal squamous cell carcinoma models (143).

Immunotherapy resistance can be promoted by CAFs through multiple mechanisms. The physical and chemical barriers established by CAFs — CXCL12-mediated T cell exclusion and NOX4-dependent fibroblast activation and ECM remodeling — can limit the efficacy of immune checkpoint inhibitors (7, 111). In mouse tumor models, using linsitinib to target the IGF2/IGF1R signaling pathway reduced CAF-mediated CD8^+^ T cell exclusion and enhanced the efficacy of immunotherapy (144); NOX4 inhibition also enhanced the immunotherapy effect by increasing T cell infiltration in CAF-rich mouse tumor models (111).

#### 4.1.6 Maintenance of cancer stem cell properties

CAFs can maintain cancer stem cell (CSC) characteristics through multiple interconnected mechanisms. In bladder cancer models, chemotherapy-induced tumor-cell pyroptosis reprograms α-SMA^+^ CAFs toward collagen III-producing inflammatory states that support CSC expansion (145). Senescence-like TSPAN8^+^ myCAFs secrete IL-6 and IL-8 to amplify CSC properties, promoting chemoresistance in breast cancer through enhanced aldehyde dehydrogenase activity and self-renewal capacity (146). The miR-146a-5p derived from CAFs inhibits the expression of ARID1A and PRKAA2, leading to activation of STAT3 and mTOR signaling, respectively, thereby enhancing the stem cell characteristics and chemotherapy resistance of bladder cancer cells (147). In laryngeal cancer, the reduced abundance of miR-34c-5p in CAF-derived exosomes contributes to maintaining the stem cell-like phenotype in experimental models (148). In mouse models of triple-negative breast cancer, Hedgehog signaling reprograms CAFs to support cancer stem cell properties and chemoresistance through FGF5 expression and fibrillar collagen production (86).

### 4.2 Antitumorigenic functions of CAFs

The recognition that certain CAF states can exert tumor-restrictive effects represents an important conceptual advance in the field, with implications for therapeutic targeting (149, 150). The terms tumor-restraining CAFs (rCAFs) and tumor-promoting CAFs (pCAFs) are functional designations rather than fixed, mutually exclusive populations. In liver metastasis models, myCAF-derived type I collagen can function as a stromal “brake” by mechanically restraining tumor spread, despite the tumor-promoting effects of other CAF-derived mediators (16).

Meflin (ISLR) is a candidate marker of tumor-restraining CAFs in PDAC. In the KPC mouse model of pancreatic cancer, Meflin-positive stromal cells appear around acinar-to-ductal metaplasia and preinvasive lesions (74). In a human PDAC cohort, the infiltration of Meflin^+^ CAFs was significantly associated with a favorable prognosis for patients (74). Lineage tracing showed that Meflin-positive cells can give rise to α-SMA^+^ CAFs with or without Meflin expression (74). It is noteworthy that the expression of Meflin is upregulated by the vitamin D analog calcipotriol in cultured pancreatic stellate cells (74). Together with evidence that vitamin D receptor activation promotes a more quiescent stellate-cell state, this finding supports a preclinical rationale for stromal reprogramming (151).

ifCAFs have demonstrated tumor-restraining properties in experimental PDAC models. Profiling of FAP^+^ stromal cells in PDAC identified an interferon-responsive subtype (58). Mechanistically, ifCAFs can suppress tumor cell invasiveness and promote an antitumor phenotype in tumor-associated neutrophils. Moreover, STING agonists can promote an ifCAF phenotype both *in vitro* and *in vivo*, supporting further preclinical investigation of this reprogramming strategy (58). A higher proportion of complement-secreting CAFs (csCAFs) has been associated with a favorable prognosis in PDAC (152). Whether this association reflects a causal contribution of CAF-derived complement to anti-tumor immunity remains unresolved.

### 4.3 Implications of functional duality for therapeutic development

In the same microenvironment, CAFs with tumor-promoting and tumor-suppressing activities can coexist, which has profound therapeutic significance. Non-specifically eliminating CAFs may disrupt protective stromal functions that physically limit tumor spread and support anti-tumor immunity. This concern has been demonstrated in PDAC mouse models: when α-SMA^+^ myofibroblasts are depleted, a more invasive and poorly differentiated tumor phenotype and immunosuppression occur, with reduced survival (18). In another study, Hedgehog pathway inhibition with the SMO antagonist LDE225 reduced short-term tumor growth in KPC mice, while decreasing myCAF abundance and expanding iCAFs alongside increased immunosuppressive Treg infiltration (87). These preclinical findings highlight a crucial principle: therapeutic outcomes depend on the stromal functions affected and tumor type, disease stage, and treatment exposure. These observations support a precise targeted strategy of preferentially inhibiting tumor-promoting CAF functions while retaining or even enhancing tumor-restrictive activities. Achieving this selectivity requires a deeper understanding of the molecular mechanisms governing these functional differences and the development of therapeutic tools capable of selectively modulating these functions.

## 5 Therapeutic strategies targeting CAFs

The therapeutic strategies targeting CAFs can be classified into four main categories: direct depletion of CAF populations, targeting the ECM components produced by CAFs, disrupting CAF-tumor signaling crosstalk, and phenotypic normalization (**Table 3**; **Figure 4**). The following sections review the rationale and evidence for these approaches, distinguishing preclinical findings from clinical outcomes and considering target selectivity, variation in effects across tumors and treatments, and barriers to translation.

**Table 3:** Therapeutic approaches targeting CAFs: strategy matrix.

Strategy category	Agent or intervention	Mechanism	Study setting and design	Key findings	Major limitations
Direct targeting and depletion	Sibrotuzumab (anti-FAP)	Antibody binding to FAP on stromal cells	Early, uncontrolled Phase II trial; mCRC; 25 enrolled, 17 evaluable	0% ORR among evaluable patients (153)	Small, uncontrolled study; CAF depletion and antitumor efficacy were not demonstrated
	^177^Lu-LNC1004 (^177^Lu-EB-FAPI)	FAP-targeted radionuclide delivery	Phase I; first-in-human, nonrandomized dose escalation; metastatic radioiodine-refractory thyroid cancer; n=12	25% ORR (3/12), 83% DCR (10/12) (154)	Small, uncontrolled study; grade 3–4 hematotoxicity in three patients; selective CAF depletion was not established
	FAP-CAR-T	CAR-T killing of FAP^+^ stromal cells	Preclinical; syngeneic mouse tumor models using different CAR constructs	73.3-FAP-CAR reduced FAP-high stroma and tumor growth, with minimal off-tumor toxicity in the tested models (155)	FAP5-CAR caused cachexia and bone toxicity in other mouse models; safety is construct- and model-dependent (156)
	ABT-199 (venetoclax) (senolytic)	BCL-2 inhibition in senescent CAFs	Preclinical; mouse PDAC; combined with anti-PD-1, anti-CTLA-4 and agonistic anti-CD40	Increased activated CD8^+^ T cells; the combination reduced tumor burden (157)	Targets a senescent CAF subset; systemic BCL-2 inhibition is not CAF-specific; clinical benefit remains unestablished
ECM modification	PEGPH20 (hyaluronidase)	HA degradation	Phase Ib/randomized II; SWOG S1313; untreated metastatic PDAC; PEGPH20 + mFOLFIRINOX versus mFOLFIRINOX	Median OS 7.7 versus 14.4 months; increased grade 3–4 treatment-related toxicity (158)	Stopped early for futility; inferior survival with this combination
			Randomized, placebo-controlled Phase III HALO 301; HA-high metastatic PDAC; PEGPH20 + GA versus placebo + GA	no OS benefit (11.2 versus 11.5 months; HR 1.00); no PFS improvement (159)	HA selection did not establish survival benefit; some severe adverse events were more frequent
	YAP1 silencing	YAP1-dependent CAF state reprogramming	Preclinical; prostate cancer organoid and mouse models	Promoted lymphocyte-associated CAF states, CD8^+^ T-cell infiltration and anti-PD-1 activity (160)	Genetic perturbation evidence; clinical benefit and effects of pharmacological YAP/TAZ inhibitors cannot be inferred
ECM modification	losartan (ARB)	Angiotensin II type 1 receptor blockade	Single-arm Phase II; LAPC; n=49; FOLFIRINOX + losartan followed by CRT	69.4% resection rate (34/49); R0 resection in 30/49 (61%) (161)	No concurrent control; losartan’s independent contribution cannot be separated from the combined treatment
Signaling disruption	Bintrafusp alfa (M7824)	Dual TGF-β + PD-L1 blockade by a bifunctional fusion protein	Single-arm Phase II; recurrent/metastatic nasopharyngeal cancer; n=38	23.7% ORR (162)	No randomized comparator; hyperprogression and adverse-event-related discontinuations raised concerns
			Single-arm Phase II; previously treated advanced biliary tract cancer; n=159	10.7% ORR; the prespecified primary endpoint was not met (163)	Uncontrolled activity does not establish comparative benefit; cancer-specific results require separate interpretation
			Randomized Phase III; treatment-naive, PD-L1-high advanced NSCLC; versus pembrolizumab; n=304	No superiority in PFS or OS; stopped following interim futility assessment (164)	Grade 3–4 treatment-related adverse events were more frequent than with pembrolizumab
	AMD3100 (plerixafor) (CXCR4 antagonist)	CXCL12-CXCR4 axis interruption to reduce T-cell exclusion	Preclinical; autochthonous KPC mouse PDAC model	AMD3100 + anti-PD-L1 synergy; reduced cancer-cell burden (7)	Model-specific evidence; clinical efficacy was not evaluated
	anakinra (IL-1Ra)	IL-1 receptor blockade; modulation of inflammatory CAF polarization and irradiation-induced senescence	Preclinical; murine rectal cancer with irradiation	Improved radiation response in mice (165)	Preclinical results do not establish patient benefit
			Phase I trial protocol; rectal cancer; anakinra + capecitabine-based CRT	Protocol published; combination tolerability is the primary objective (166)	The protocol provides no patient efficacy results
Phenotypic normalization	Calcipotriol (VDR agonist)	PSC quiescence induction through VDR activation	Preclinical; PSC cultures and mouse PDAC models; combined with gemcitabine	Increased lipid accumulation; enhanced gemcitabine efficacy and survival in mice (151)	Preclinical therapeutic benefit; patient efficacy remains unestablished
	paricalcitol (VDR agonist)	VDR-mediated stromal and immune modulation	Pilot study; nonrandomized; advanced PDAC after gemcitabine; n=20; IV paricalcitol + nal-IRI/5-FU/LV	Increased CD31 staining in three paired biopsies in the higher-dose cohort (167)	Small, uncontrolled study; vascular-marker changes do not establish improved perfusion or clinical efficacy
Phenotypic normalization	paricalcitol (VDR agonist)	VDR-mediated stromal and immune modulation	Randomized three-arm run-in trial; metastatic PDAC; n=36; GA + placebo, IV paricalcitol or oral paricalcitol	Biopsies showed a lower proportion of α-SMA^+^ fibroblasts and greater CD8^+^ T-cell density and proximity to cancer cells (168)	Safety-focused study; clinical efficacy unestablished; hypercalcemia required dose reduction in the oral arm
			Single-arm phase II; previously untreated advanced PDAC; n=15; oral paricalcitol + GA	Prespecified 6-month PFS threshold not met; stopped after interim analysis (169)	Small, uncontrolled study; benefit unestablished; hypercalcemia included grade 3 events
	ATRA (RAR agonist)	PSC quiescence induction and mechanical reprogramming	Preclinical; PSC cultures and KPC mouse PDAC models	Reduced matrix remodeling and tumor invasion; reduced paracrine Wnt–β-catenin signaling and tumor progression (170, 171)	Model-dependent effects; clinical benefit cannot be inferred from these experiments
			Nonrandomized phase Ib; advanced PDAC; n=27; ATRA + GA	Established a recommended combination dose and provided exploratory evidence of stromal modulation (172)	No randomized efficacy comparison; dose-limiting thrombocytopenia occurred
	tranilast polymeric micelles	Antifibrotic stromal reprogramming with enhanced intratumoral drug delivery	Preclinical; mouse breast cancer; combined with nanochemotherapy and anti-PD-1/anti-CTLA-4	Reduced tumor stiffness; combination treatment enhanced T-cell infiltration and durable tumor control (173)	Formulation- and combination-dependent findings; no human efficacy or safety data in this study

Strategy categories overlap mechanistically. Preclinical effects and pharmacodynamic changes do not establish clinical benefit. Outcomes of combination regimens cannot be attributed to the CAF-directed component alone. FAP expression is not restricted to tumor CAFs, and several interventions also act on non-CAF compartments.

ARB, angiotensin receptor blocker; ATRA, all-trans retinoic acid; BCL-2, B-cell lymphoma 2; CAF, cancer-associated fibroblast; CAR-T, chimeric antigen receptor T cell; CRT, chemoradiotherapy; CTLA-4, cytotoxic T-lymphocyte-associated protein 4; CXCL12, C-X-C motif chemokine ligand 12; CXCR4, C-X-C motif chemokine receptor 4; DCR, disease control rate; ECM, extracellular matrix; FAP, fibroblast activation protein; GA, gemcitabine plus nab-paclitaxel; HA, hyaluronan; HR, hazard ratio; IL-1Ra, interleukin-1 receptor antagonist; IV, intravenous; KPC, Kras/Trp53-mutant pancreatic cancer mouse model; LAPC, locally advanced pancreatic cancer; mCRC, metastatic colorectal cancer; mFOLFIRINOX, modified fluorouracil, leucovorin, irinotecan and oxaliplatin; nal-IRI/5-FU/LV, nanoliposomal irinotecan, fluorouracil and leucovorin; NSCLC, non-small cell lung cancer; ORR, objective response rate; OS, overall survival; PDAC, pancreatic ductal adenocarcinoma; PD-1, programmed cell death protein 1; PD-L1, programmed death-ligand 1; PFS, progression-free survival; PSC, pancreatic stellate cell; R0, microscopically margin-negative resection; RAR, retinoic acid receptor; TAZ, transcriptional coactivator with PDZ-binding motif; TGF-β, transforming growth factor β; VDR, vitamin D receptor; YAP1, Yes-associated protein 1; α-SMA, α-smooth muscle actin.

**Figure 4:**
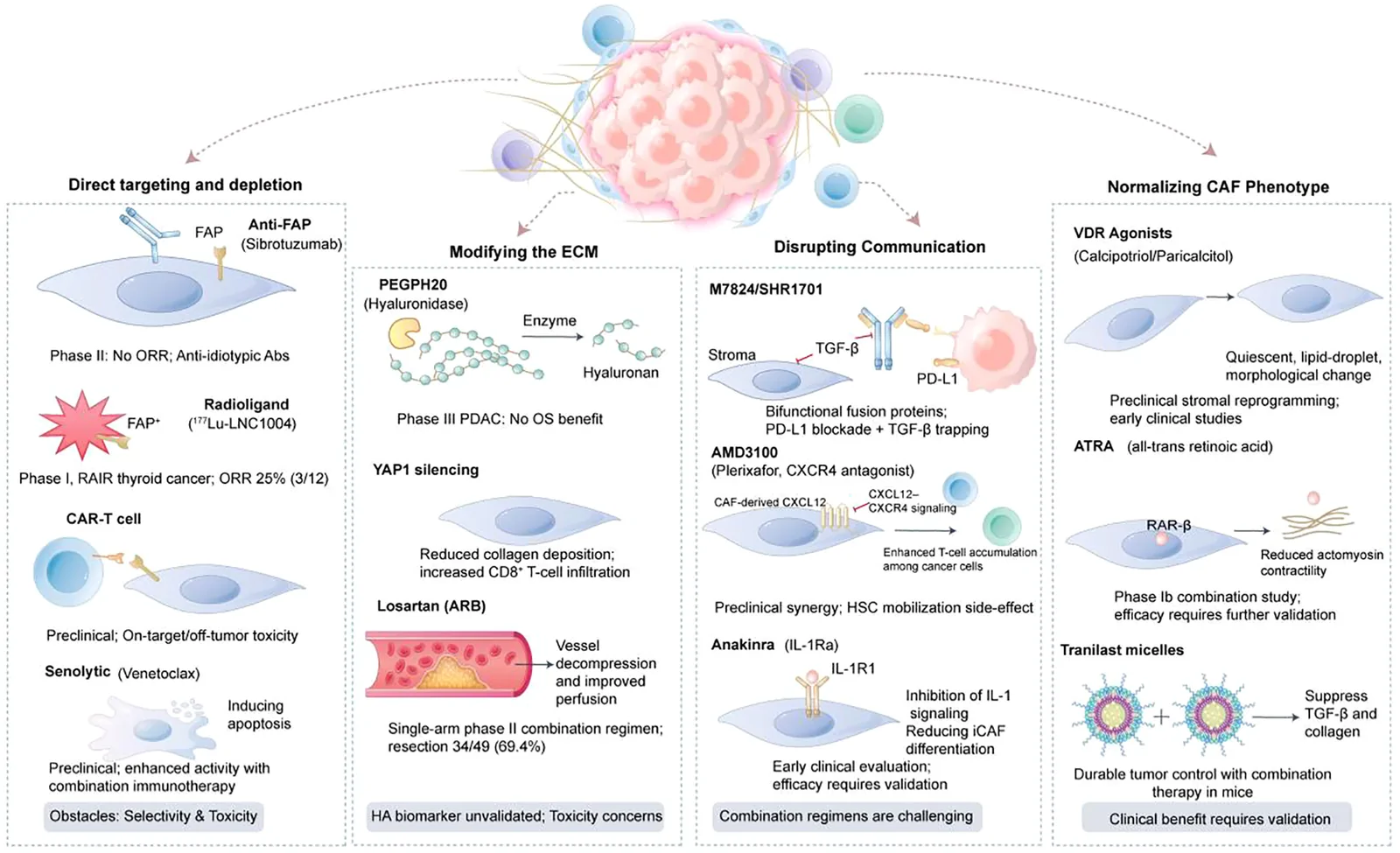
Therapeutic strategies targeting CAFs. Four strategies are illustrated with representative agents and mechanisms, including direct targeting and depletion, ECM modification, signaling disruption, and phenotypic normalization. Labels identify the study setting and reported outcomes. Normalization approaches aim to shift activated stromal programs toward a more quiescent state. Ab, antibody; ARB, angiotensin II receptor blocker; ATRA, all-trans retinoic acid; CAF, cancer-associated fibroblast; CAR-T, chimeric antigen receptor T cell; CXCL12, C-X-C motif chemokine ligand 12; CXCR4, C-X-C motif chemokine receptor 4; ECM, extracellular matrix; FAP, fibroblast activation protein; HA, hyaluronan; HSC, hematopoietic stem cell; iCAF, inflammatory CAF; IL-1, interleukin-1; IL-1Ra, interleukin-1 receptor antagonist; IL-1R1, interleukin-1 receptor type 1; ORR, objective response rate; OS, overall survival; PDAC, pancreatic ductal adenocarcinoma; PD-L1, programed death-ligand 1; PEGPH20, pegylated recombinant human hyaluronidase PH20; RAIR, radioiodine-refractory; RAR-β, retinoic acid receptor β; TGF-β, transforming growth factor β; VDR, vitamin D receptor; YAP1, Yes-associated protein 1; ^177^Lu, lutetium-177.

### 5.1 Direct CAF targeting and depletion

#### 5.1.1 FAP-targeted therapies

Fibroblast Activation Protein (FAP) has become one of the most extensively studied target molecules for CAF-directed therapy due to its expression on many CAFs, its enzymatic activity, and its cell-surface localization (153, 174). Early clinical development focused on the humanized monoclonal antibody sibrotuzumab, which targeted FAP on stromal fibroblasts. However, the phase II trial for patients with metastatic colorectal cancer reported no complete or partial responses among 17 evaluable patients (153). Subsequently, researchers explored various alternative FAP-targeting strategies. Among them, the use of Evans blue-modified FAP inhibitors labeled with therapeutic isotopes for radioligand therapy is an especially active research direction. The albumin-binding radioligand ^177^Lu-LNC1004 (^177^Lu-EB-FAPI) demonstrated preliminary antitumor activity in a nonrandomized phase I trial in 12 patients with metastatic radioiodine-refractory thyroid cancer, with an objective response rate of 25% and a disease control rate of 83% (154). Grade 3–4 hematotoxicity was observed at the higher dose levels. In a separate exploratory study, single-cell analysis of peripheral blood mononuclear cells after ^177^Lu-LNC1004 treatment showed enrichment of antigen processing and presentation gene sets, but also signatures of T cell inactivation, suggesting that further investigation of this approach in combination with immunotherapy is warranted (175). FAP-based vaccines aim to break the immune tolerance to FAP and promote the cytotoxic T cell response targeting FAP-positive CAFs. Preclinical studies have shown that vaccination slows tumor growth and enhances T cell infiltration (176). CAR-T cells targeting FAP are another investigational approach, and current preclinical models indicate that this method can disrupt the tumor stroma and restrain tumor growth (176). However, due to the risk of on-target, off-tumor toxicity (FAP is expressed on multipotent bone marrow stromal cells), careful design of CARs and enhanced safety monitoring are necessary (156). Additionally, FAP-targeted imaging using ^68^Ga-DOTA-FAPI-04 PET/MR has demonstrated superior performance over ^18^F-FDG PET/CT in detecting primary and most metastatic lesions of gastric cancer in a 20-patient study (177).

#### 5.1.2 Senolytic approaches

Senescent CAFs in PDAC exhibit altered SASP and immune-regulatory programs and can contribute to immunosuppression (157). In pancreatic cancer mouse models, the Bcl-2-selective inhibitor ABT-199 (venetoclax) depleted senescent CAFs and increased the proportion of activated CD8^+^ T cells. When combined with anti-PD-1, anti-CTLA-4, and agonistic anti-CD40 antibodies, ABT-199 further reduced tumor burden compared with immunotherapy alone (157). This method of eliminating senescent fibroblasts is a preclinical strategy for targeting immunosuppressive CAF states, but its clinical efficacy, optimal timing and patient selection still need to be established.

### 5.2 Targeting CAF-derived ECM components

#### 5.2.1 Hyaluronan degradation

The glycosaminoglycan hyaluronic acid (HA) is an ECM component produced by CAFs. Excessive accumulation of HA can contribute to increased interstitial fluid pressure and collapse of tumor blood vessels, thereby hindering drug delivery and immune cell infiltration. Pegylated recombinant human hyaluronidase (PEGPH20) can degrade HA in tumors and reduce interstitial pressure and improve vascular patency in preclinical models (178). Although it showed promising results in preclinical studies, a phase III pivotal trial (HALO-301) combining PEGPH20 with nab-paclitaxel plus gemcitabine for HA-high metastatic pancreatic cancer failed to demonstrate significant survival benefits (median overall survival, 11.2 versus 11.5 months; hazard ratio, 1.00) (159). In the randomized phase II component of SWOG S1313, adding PEGPH20 to mFOLFIRINOX in patients unselected for HA status increased toxicity and was associated with shorter overall survival than mFOLFIRINOX alone (7.7 versus 14.4 months) (158). Additionally, its clinical efficacy when used in combination with atezolizumab in previously treated advanced pancreatic cancer and gastric cancer was also limited in the randomized phase Ib/II MORPHEUS studies, with no improvement in clinical outcomes over chemotherapy (179). These results do not validate HA abundance alone as a predictive biomarker for clinical benefit from PEGPH20 (159, 179).

#### 5.2.2 Therapeutic ECM reprogramming

YAP1 inhibition is an emerging preclinical strategy that can be used to reprogram ECM-producing CAFs into an immunostimulatory phenotype. In prostate cancer models, selective depletion of YAP1 in ECM-associated CAFs promoted the nuclear translocation of NF-κB p65, promoting the recruitment and activation of CD8^+^ T cells, and reducing collagen deposition (160). Dendritic polymer prodrugs of dasatinib (P-DAS) reduce collagen synthesis by CAFs and improve the tumor penetration of sequentially administered epirubicin prodrugs (P-Epi), with this combination enhancing anti-tumor immune responses in breast cancer-bearing mice (180).

#### 5.2.3 Vascular normalization

Angiotensin II receptor blockade with losartan reduces CAF activation and collagen and hyaluronan production, thereby improving tumor vascular perfusion and reducing solid stress in breast and pancreatic cancer models (181). A single-arm phase II trial of losartan plus FOLFIRINOX chemotherapy followed by chemoradiation in locally advanced PDAC reported rates of surgical resection (34/49; 69.4%) and R0 resection (30/49; 61%) (161). The absence of a concurrent control group prevents attribution of these outcomes to losartan alone.

### 5.3 Disrupting CAF-tumor signaling crosstalk

#### 5.3.1 TGF-β pathway inhibition

Given the central role of TGF-β signaling in fibroblast activation and immunosuppression, various inhibitors have been developed and used in clinical trials. A dual-function fusion protein, bintrafusp alfa (M7824), which can simultaneously block TGF-β and PD-L1, has shown modest activity in single-arm phase II studies of nasopharyngeal and biliary tract cancers (162, 163). However, in a randomized phase III clinical trial in PD-L1-high advanced non-small cell lung cancer, the efficacy of this drug was not superior to that of pembrolizumab, and grade 3–4 treatment-related adverse events were more frequent (164). Another dual-functional fusion protein, SHR-1701, when used in combination with neoadjuvant chemotherapy, showed antitumor activity in a proof-of-concept phase II trial for unresectable stage III non-small cell lung cancer, with an objective response rate of 58.0% after induction in the 97-patient combination cohort (182). These mixed results support further evaluation of the selection criteria of patients, the timing of treatment, and the combined strategy in controlled studies.

#### 5.3.2 Immune modulation and stromal reprogramming

The use of anakinra to block the IL-1 receptor has been shown to increase CD8^+^ T cells in murine rectal tumors after irradiation (165). Its combined application with capecitabine-based chemotherapy and radiotherapy was described in a published Phase I trial protocol for rectal cancer (166). In mouse PDAC models, inhibition of Hedgehog signaling with sonidegib (LDE225) altered the composition of CAF subgroups, reducing myCAFs and enriching iCAFs, alongside decreased CD8^+^ T-cell infiltration (87).

### 5.4 Phenotypic normalization

Rather than eliminating CAFs, which risks removing potentially protective populations, the strategy of inducing CAF quiescence or normalization aims to shift activated CAFs toward a resting state, with the goal of preserving stromal integrity while reducing protumorigenic functions. This approach remains experimental and requires further clinical validation.

#### 5.4.1 Vitamin D receptor agonists

VDR agonists are an experimental normalization strategy for CAFs evaluated in early clinical studies. In human PDAC, cancer-associated pancreatic stellate cells express high levels of VDR. Treatment with calcipotriol (a VDR agonist) can induce a quiescent phenotype, characterized by lipid droplet accumulation, reduced α-SMA expression, and decreased extracellular matrix (ECM) production in preclinical models (151). The synthetic vitamin D analog calcipotriol significantly improves the efficacy of gemcitabine in mouse PDAC models by improving vascular patency and drug delivery, thereby increasing the concentration of the active metabolite dFdCTP within the tumor (151). A preliminary nonrandomized, 20-patient study suggests that a higher dose of paricalcitol (7 mcg/kg/week) combined with Nal-IRI and 5-FU/LV has good tolerability in patients with advanced PDAC after progression on gemcitabine-based therapy; increased CD31 staining in paired biopsies from three patients suggested an effect on tumor vascularity, without establishing improved clinical efficacy (167). A randomized, 36-patient run-in trial of paricalcitol plus gemcitabine/nab-paclitaxel demonstrated stromal and immune modulation in paired biopsies, but was not powered to establish clinical efficacy (168). A separate 15-patient, single-arm phase II trial of oral paricalcitol with the same chemotherapy backbone stopped early after failing to meet its prespecified efficacy threshold (169). In addition to pancreatic cancer, in preclinical gastric cancer models, VDR activation in cancer cells reverses oxaliplatin resistance mediated by IL-8 derived from CAFs by inhibiting the PI3K/Akt signaling pathway (183). This finding reflects modulation of cancer-cell responses to CAF-derived signals and does not demonstrate direct CAF normalization.

#### 5.4.2 Retinoic acid receptor agonists

All-trans retinoic acid (ATRA) downregulates the expression of myosin light chain 2 (MLC2) through the retinoic acid receptor-β (RAR-β), reduces actomyosin contractility, inhibits the mechanosensory activation of pancreatic stellate cells, and thereby reduces the matrix remodeling ability *in vitro* (170). In the KPC pancreatic cancer model, ATRA can reduce the activation of PSCs, interfere with the Wnt-β-catenin signaling pathway in cancer cells, and inhibit cancer cell proliferation (171).

A nonrandomized phase Ib trial in 27 patients supported the safety and tolerability of the combination of ATRA with gemcitabine and nab-paclitaxel. Higher chemotherapy dose intensity than in a historical trial and apparently reduced neurotoxicity were observed, but require confirmation in randomized studies (172).

#### 5.4.3 Other normalization strategies

Beyond VDR and RAR agonists, additional approaches for CAF reprogramming are under investigation. Tranilast, an anti-allergic medication with anti-fibrotic properties, loaded into polymeric micelles has shown preclinical activity, diminishing tumor stiffness and, when combined with epirubicin micelles or liposomal doxorubicin plus anti-PD-1/anti-CTLA-4 antibodies, amplifying T cell recruitment and producing durable remission in immunotherapy-resistant mouse breast cancer models (173).

The observation that Meflin expression is upregulated by the vitamin D analog calcipotriol in cultured PSCs suggests that VDR agonists may modulate CAF functional states (73). This concept of “dual normalization”, simultaneously suppressing protumorigenic functions while enhancing tumor-restraining functions, remains a hypothesis requiring functional and clinical validation.

### 5.5 Clinical challenges and barriers to translation

The current strategies targeting CAFs have therapeutic potential, but the clinical translation of related methods still faces several major challenges. Target selectivity remains problematic: markers such as FAP and CD10 are expressed not only on CAFs but also on certain normal stromal or immune cells and, in some cases, cancer cells themselves, potentially leading to on-target, off-tumor toxicity and misclassification (184–186). FAP expression in bone marrow stromal cells raises particular concerns for CAR T cell approaches due to on-target, off-tumor toxicity observed in preclinical models (156).

CAF heterogeneity and plasticity complicate therapeutic precision. The coexistence of multiple CAF subtypes within individual tumors and their ability to interconvert in response to therapy represent formidable obstacles. Chemoresistance-inducing CD10^+^GPR77^+^ CAFs can be activated through TAM-secreted CCL18 in breast cancer models, supporting investigation of subset-specific targeting strategies (187).

Preclinical studies illustrate potential compensatory signaling and stromal adaptation. During colitis-associated tumorigenesis in mice, Col6a1^+^ CAFs partly retain homeostatic characteristics while acquiring a pro-angiogenic transcriptional signature. However, separate genetic deletion of TNFR1 or IL-1R1 in Col6a1^+^ cells did not significantly reduce tumor number. Possible explanations include redundant inflammatory signals and compensatory activation of other CAF subpopulations (188). In an EGFR-mutant lung cancer xenograft model, erlotinib treatment shifted CAFs toward an IL-6-producing state, accompanied by increased NF-κB/STAT3 signaling in drug-tolerant persister cells (189). These adaptive responses support further investigation of multi-target combination strategies.

The dynamic nature of CAF phenotypes supports longitudinal therapeutic monitoring. The use of ^68^Ga-FAPI PET/CT for non-invasive imaging of FAP^+^ stroma represents a promising approach, with diagnostic data linking tracer uptake to stromal FAP expression in mucoepidermoid carcinoma (190). Its utility for assessing CAF-directed therapy efficacy and guiding treatment sequencing remains to be validated.

## 6 Future perspectives

### 6.1 Single-cell and spatial multiomics integration

The continuous optimization of multi-omics technologies is expected to further reveal the heterogeneity of CAFs and guide the formulation of treatment strategies. The combination of multi-omics can map the distribution of CAF subtypes in the original tissue structure and infer candidate intercellular interactions (14, 191, 192). In hepatocellular carcinoma, spatial multi-omic profiling distinguished intratumoral FAP/POSTN-enriched CAFs from peri-tumoral C7/PDGFRA-enriched CAFs, each occupying distinct microdomains with different immune neighborhoods and opposing associations with patient survival (193).

Demonstrated computational applications include CAF classification (14), prognostic association analysis (192), and deep-learning quantification of stromal content and immune infiltration in H&E images (193). Machine learning and artificial intelligence approaches applied to multiomic datasets could be developed to integrate CAF subtype composition with genomic, transcriptomic, and clinical data to predict prognosis, identify therapeutic vulnerabilities, and forecast compensatory pathway activation. Such approaches may ultimately guide rational combinatorial regimen design by modeling CAF-tumor-immune signaling networks, but predicting treatment-induced adaptation and clinical benefit requires longitudinal data, independent validation, and prospective testing (**Table 4**).

**Table 4:** Computational and AI approaches in CAF research: current evidence and future directions.

Application domain	Methods & tools	Evidence in CAF research	Key limitation	Future directions
CAF subtype discovery	scRNA-seq clustering; marker-based annotation; imaging mass cytometry	CAF states identified in breast cancer and evaluated in additional cancer types, with protein-level and spatial validation in matched breast tumors (14)	Batch effects; annotation standards lacking; rare subtypes missed at low coverage	Harmonized CAF-state annotation and improved reproducibility across studies
Spatial mapping and stromal quantification	Spatial transcriptomics; spatial proteomics; H&E deep learning	Spatial multiomics maps CAF localization; H&E deep learning quantifies stromal content and immune infiltration in hepatocellular carcinoma (193)	Platform-dependent resolution and coverage; H&E-based assignment of molecular CAF states requires matched molecular validation	Joint analysis of spatial omics and histology to connect stromal architecture with molecularly defined CAF states
Prognostic modeling	CAF signature scoring and Cox regression	CAF-related molecular and spatial features associated with outcomes in retrospective chordoma and hepatocellular carcinoma cohorts (192, 193)	Model generalizability across platforms; limited prospective validation	Independent and prospective validation of CAF-associated prognostic models
Drug response prediction	Proposed integration of multiomic and drug-response data	The studies reviewed here do not establish validated prediction of response to TGF-β, VDR, or STING modulators	Limited paired patient samples with both multiomics and drug response	Develop and independently validate response models using paired molecular and treatment-response data
Candidate resistance mechanism inference	CellChat; pathway analysis of treatment-associated transcriptomic changes	Candidate intercellular signals inferred in chordoma; treatment-associated CAF-state shifts observed in an EGFR-mutant lung cancer xenograft model (189, 192)	Predictions require wet-lab validation; dynamic signaling not captured by static profiling	Temporal signaling models integrating longitudinal biopsies and perturbation experiments to track CAF state evolution under therapy
Combinatorial regimen design	Proposed computational prioritization of drug combinations	Preclinical combination studies discussed in this review provide a rationale for modeling but do not establish AI-guided regimen design	Combinatorial explosion; limited CAF-focused perturbation data; proposed combinations require experimental validation	Prioritize candidate combinations for testing in CAF-containing models and subsequent prospective studies

Current applications include CAF classification, spatial quantification, and prognostic analysis. Treatment-response prediction and computational regimen design are presented as future directions.

AI, artificial intelligence; CAF, cancer-associated fibroblast; EGFR, epidermal growth factor receptor; H&E, hematoxylin and eosin; scRNA-seq, single-cell RNA sequencing; STING, stimulator of interferon genes; TGF-β, transforming growth factor β; VDR, vitamin D receptor.

### 6.2 Patient-derived models for drug development

Patient-derived organoids, xenografts, and three-dimensional co-culture systems containing CAFs provide complementary platforms for studying drug responses (194–197). Standard epithelial organoids do not automatically retain native CAFs or immune cells, while conventional xenografts generally depend on host-derived stroma and immunodeficient mice (196, 197). Introducing CAFs into the culture system of patient-derived organoids can reproduce selected key features of the TME, including CAF-mediated changes in drug responses (198). Combining patient-derived models with single-cell and spatial omics technologies may help to reveal changes in CAF behavior induced by treatment and identify candidate resistance mechanisms, such as treatment-associated signaling changes (189). However, the scaling up of these systems for high-throughput screening applications still faces technical challenges, and standardization protocols among laboratories need to be improved to account for culture-dependent variation in drug responses (196).

### 6.3 Combinatorial regimens

Given the inherent complexity and adaptive capacity of CAFs, effective therapeutic strategies will likely require multi-pronged approaches. Dual inhibition of complementary signaling pathways may block compensatory activation; for example, combined TGF-β and CXCR4 inhibition could be tested to simultaneously address CAF activation and immune exclusion. The combined use of CAF regulators and immune checkpoint inhibitors may reduce stromal immune suppression. Preclinical evidence includes depletion of senescent CAFs with ABT-199 combined with anti-PD-1/anti-CTLA-4 and agonistic anti-CD40 treatment in PDAC mouse models (157). In humans, the combination of drugs for eliminating senescent cells (dasatinib and quercetin) with anti-PD-1 therapy showed preliminary activity in a phase II study of head and neck squamous cell carcinoma, although CAF-specific effects were not established (199). Preclinically, the combination of anti-PD-1 therapy with FAP-targeted radiotherapy showed antitumor activity in mice bearing fibrosarcomas engineered to express FAP (200). Furthermore, combining drugs that interfere with ECM with chemotherapy or anti-angiogenic therapy is also a reasonable next step. Subsequent studies need to conduct systematic evaluations of these combinations and use appropriate clinical trial designs and reliable *in vitro* models.

### 6.4 Adaptive trial designs with liquid biopsy monitoring

Incorporating stromal monitoring through liquid biopsy into the design of clinical trials could support longitudinal assessment of the pharmacodynamic effects of CAF-targeted treatment. For instance, circulating cytokines, chemokines, ECM fragments, and exosomal miRNAs may serve as candidate biomarkers, but their cellular sources and relationships to treatment benefit require validation. In a randomized phase II PDAC trial, paired tumor and plasma analysis demonstrated TGF-β pathway inhibition and stromal remodeling with NIS793, without improved clinical outcomes (201). Such pharmacodynamic changes should therefore not be equated with clinical benefit. In future adaptive trials, stratification based on the baseline CAF state composition of patients could be evaluated prospectively. After using drugs targeting CAFs for treatment, the changes in CAF states and the activation of candidate resistance pathways could be assessed using paired tissue and blood samples. Any biomarker-guided treatment adjustments should follow prespecified decision rules and be tested against clinical efficacy and safety outcomes.

### 6.5 The path toward precision stromal medicine

The ultimate goal of CAF-targeted therapy is to achieve precision stromal medicine. The realization of this concept requires: (1) a clinically implementable CAF state classification system based on accessible biomarkers (immunohistochemistry, circulating markers, imaging) and validated for defined tumor types, disease stages, and treatments; (2) validated diagnostic tools for patient stratification; (3) a series of drugs with selective effects on CAF functions while preserving beneficial stromal activities; (4) treatment algorithms considering the plasticity and compensatory response of CAFs. Moreover, continuous collaboration among basic scientists, translational researchers, and clinical oncologists is needed to develop prospectively specified trial designs that combine stromal pharmacodynamic endpoints with clinical efficacy and safety outcomes.

## 7 Concluding remarks

CAFs can promote or restrain cancer progression, influencing the remodeling of the extracellular matrix, immune regulation, metabolic coupling, angiogenesis, and treatment resistance. Their cellular origins, phenotypic states, and tumor-promoting or tumor-restraining functions should be distinguished rather than treated as interchangeable classifications. The rationale for moving from large-scale stromal depletion strategies to selective modulation reflects our evolving understanding of the complexity of tumor stroma. For example, depletion of α-SMA^+^ myofibroblasts accelerated tumor progression and reduced survival in PDAC mouse models. These findings highlight the importance of preserving tumor-restraining stromal functions when developing CAF-directed therapies. Currently, CAF-directed therapies are not yet established standards of cancer care, and their clinical benefit requires further validation. Through the integration of technologies such as single-cell and spatial multi-omics, patient-derived organoids and co-culture platforms, our understanding of CAF heterogeneity and plasticity has advanced. The clinical value of artificial intelligence-based predictive modeling requires independent and prospective validation. Integrating CAF classification into clinical workflows will require standardized assays and prospective evidence of clinical utility to support the vision of precision stromal oncology.
